# The Best Preliminary Education for the Study of Medicine

**Published:** 1886-10

**Authors:** 


The Best Preliminary Education for the Study of Medicine. *By* Edward S. Stevens, m. d. *Being the* Boylston Prize Essay, 1885. *The Western Star Print, Lebanon, Ohio.*

The opening sentence of this work reads: "This essay will be written from an American standpoint." It would have been more correct to have said, this essay will be written from the standpoint of one individual American, and addressed to a select *coterie* of medical gentlemen in an eastern city. In marked contrast to the narrowness of this work is the comprehensive view taken of the subject of American medical education in the address delivered before the British Medical Association at its last annual meeting by Dr. J. S. Billings.

Incidentally, we may express a doubt as to the general utility of prize essays, and of this one in particular.

MEDICINE OF THE FUTURE. *An Address prepared for the Annual Meeting of the British Medical Association in 1886, by* AUSTIN FLINT (SENIOR), M.D., LL.D. *New York:* D. APPLETON & CO. *Chicago:* A. C. MCCLURG & CO.

This memorial volume is beautifully issued by the Appletons, on extra heavy paper with wide margins and double leaded lines. An excellent autotype of the author faces the title page.

The address itself must excite widespread attention, as the last work of one of the most commanding figures in American medicine.

